# Supplementary figures and images for: Effects of mucus trail following on the distance between individuals of opposite sex and its influence on the evolution of the trait in the Ezo abalone Haliotis discus hannai
Source: PeerJ. 2020 Mar 10;8:e8710. doi: 10.7717/peerj.8710 (PMC7069403; doi:10.7717/peerj.8710)

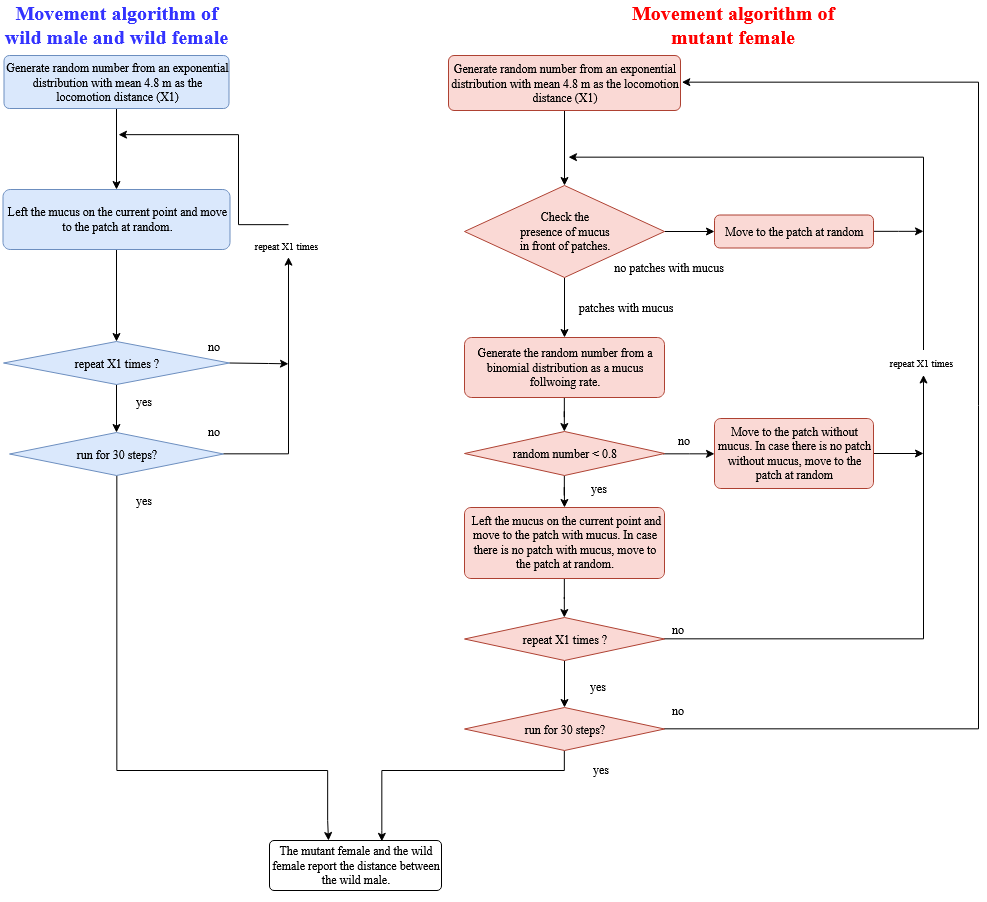

Supplement: Figure S1 — Movement algorithm in the IBM. [file peerj-08-8710-s001.png]

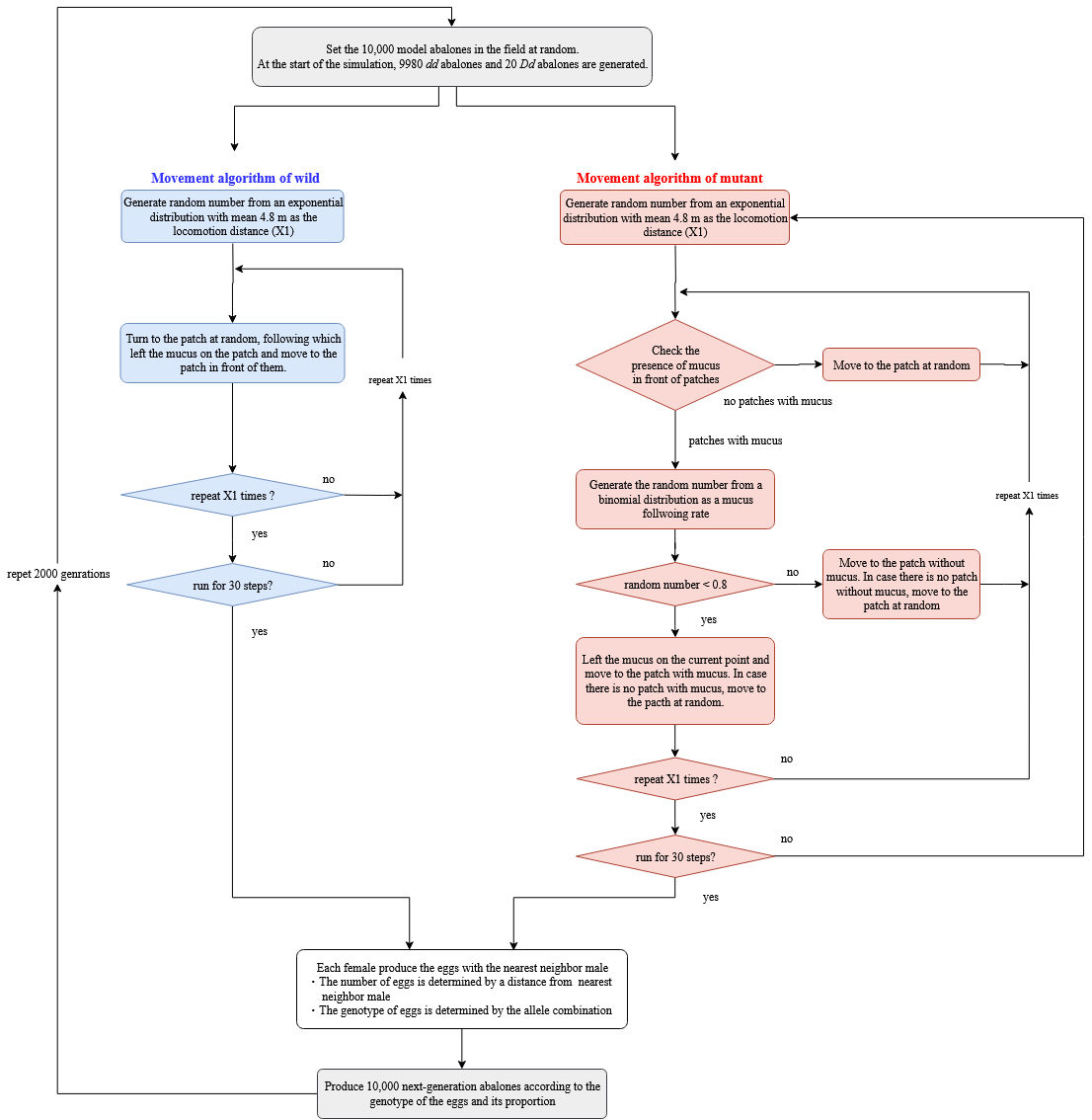

Supplement: Figure S2 [file peerj-08-8710-s002.png]

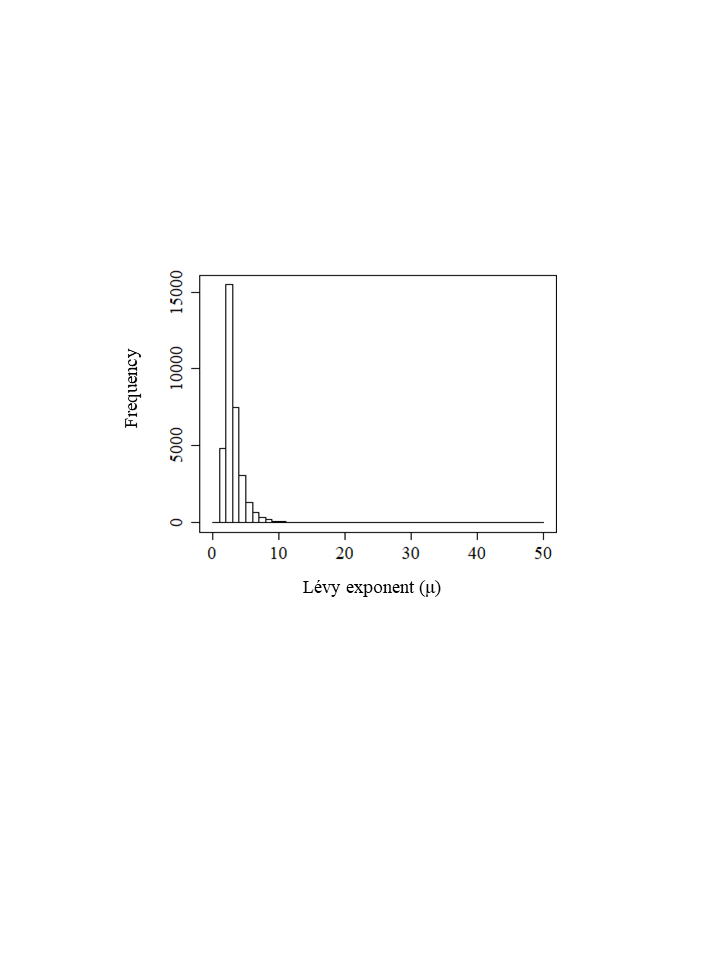

Supplement: Figure S3 — When 1 >μ ≤ 3, the locomotion pattern consist with lévy flight ※. Lévy exponent was calculated using fit_power_law function in igraph package R v3.5.2. ※H. Malchow, A. James, and R. Brown, “ Lévy or Not? Analysing Positional Data from Animal Movement Paths,” B. Dispersal, Individ. Mov. Spat. Ecol. A Math. Perspect. Springer-Verlag, vol. 2071, pp. 293-305, 2013. [file peerj-08-8710-s003.png]
