# Supplementary material for: Effects of mucus trail following on the distance between individuals of opposite sex and its influence on the evolution of the trait in the Ezo abalone Haliotis discus hannai
Source: PeerJ. 2020 Mar 10;8:e8710. doi: 10.7717/peerj.8710 (PMC7069403; doi:10.7717/peerj.8710)
Supplement: Supplemental Data S2 [file peerj-08-8710-s005.pptx]

## Slide 1
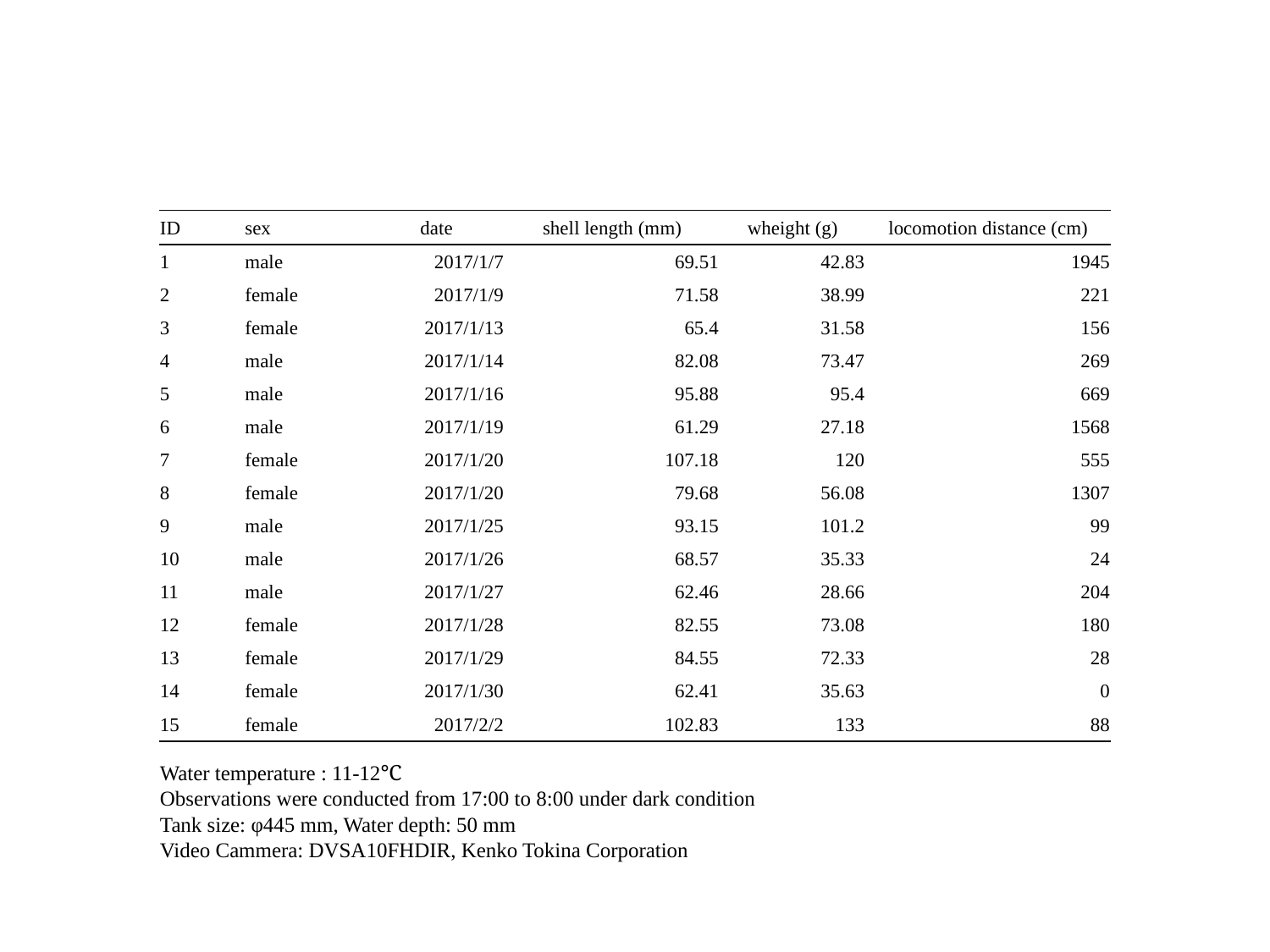

| | | | | | |
| --- | --- | --- | --- | --- | --- |
| ID | sex | date | shell length (mm) | wheight (g) | locomotion distance (cm) |
| 1 | male | 2017/1/7 | 69.51 | 42.83 | 1945 |
| 2 | female | 2017/1/9 | 71.58 | 38.99 | 221 |
| 3 | female | 2017/1/13 | 65.4 | 31.58 | 156 |
| 4 | male | 2017/1/14 | 82.08 | 73.47 | 269 |
| 5 | male | 2017/1/16 | 95.88 | 95.4 | 669 |
| 6 | male | 2017/1/19 | 61.29 | 27.18 | 1568 |
| 7 | female | 2017/1/20 | 107.18 | 120 | 555 |
| 8 | female | 2017/1/20 | 79.68 | 56.08 | 1307 |
| 9 | male | 2017/1/25 | 93.15 | 101.2 | 99 |
| 10 | male | 2017/1/26 | 68.57 | 35.33 | 24 |
| 11 | male | 2017/1/27 | 62.46 | 28.66 | 204 |
| 12 | female | 2017/1/28 | 82.55 | 73.08 | 180 |
| 13 | female | 2017/1/29 | 84.55 | 72.33 | 28 |
| 14 | female | 2017/1/30 | 62.41 | 35.63 | 0 |
| 15 | female | 2017/2/2 | 102.83 | 133 | 88 |
| | | | | | |
| Water temperature : 11-12℃ |
| --- |
| Observations were conducted from 17:00 to 8:00 under dark condition |
| Tank size: φ445 mm, Water depth: 50 mm |
| Video Cammera: DVSA10FHDIR, Kenko Tokina Corporation |
